# Translational Research with FUS BBBO and Neuromodulation for AD

**DOI:** 10.1002/alz70859_103277

**Published:** 2025-12-25

**Authors:** Jürgen Götz

**Affiliations:** ^1^ University of Queensland, Brisbane Australia

## Abstract

**Background:**

Existing therapies of Alzheimer’s disease (AD) offer only modest symptomatic benefits, and the recently approved anti‐amyloid‐β antibodies still need to demonstrate their real‐world effectiveness, warranting alternative strategies.

**Methods:**

We explore complementary treatment strategies targeting amyloid and tau deposition along with the ensuing cognitive impairment. This includes the development of novel anti‐tau antibodies and low‐intensity ultrasound used (i) as blood‐brain barrier (BBB)‐opening tool for focused intracerebral drug delivery and (ii) for neuromodulation.

**Results:**

We tested our novel anti‐ tau antibody, RNJ1, in seeding assays and in vivo and found that it outperformed HJ8.5 (tilavonemab) which was included for benchmarking. Importantly, we revealed proteostasis restoration as a new metric for tau immunotherapy efficacy. We further used low‐intensity ultrasound in amyloid‐depositing APP23 mice and found that repeated scanning ultrasound (SUS) treatment without opening the blood‐brain barrier (BBB) ameliorated memory deficits in the APP23 mouse model of AD, without reducing amyloid‐β burden. We also developed a suite of nanobodies targeting the BBB protein JAM‐1, two of which weaken the BBB aimed at facilitating drug delivery to the brain. Together, these finding formed the basis of building an ISO13485‐certified investigational UltraThera Pilot device and conducting and completing a safety trial in 12 AD patients, demonstrating safety, tolerability and feasibility.

**Conclusion:**

Our data suggest a dissociation of cognitive improvement and Aβ clearance, with important implications for the design of ultrasound trials for AD therapies. Our work further identifies a new metric for determining the efficacy of immunotherapies targeting the brain.

